# 支气管肺泡灌洗液CD4^+^/CD8^+^联合nCD64指数对肺癌与非肺癌疾病的鉴别诊断价值

**DOI:** 10.3779/j.issn.1009-3419.2026.106.08

**Published:** 2026-03-20

**Authors:** Xuexue WU, Chan YANG, Lili JIANG

**Affiliations:** 610041 成都，四川大学华西医院病理科; Department of Pathology, West China Hospital, Sichuan University, Chengdu 610041, China

**Keywords:** 支气管肺泡灌洗液, T淋巴细胞亚群, CD4^+^/CD8^+^比值, nCD64指数, 肺肿瘤, 肺感染性疾病, 鉴别诊断, Bronchoalveolar lavage fluid, T lymphocyte subsets, CD4^+^/CD8^+^ ratio, nCD64 index, Lung neoplasms, Pulmonary infection, Differential diagnosis

## Abstract

**背景与目的** 肺癌与肺感染性疾病、间质性肺疾病（interstitial lung disease, ILD）的临床鉴别尚存在困难，本研究旨在探讨支气管肺泡灌洗液（bronchoalveolar lavage fluid, BALF）中T淋巴细胞亚群及中性粒细胞CD64（neutrophil CD64, nCD64）指数单独及联合检测在上述疾病中的鉴别诊断价值。** 方法** 收集2021年10月至2022年10月于四川大学华西医院就诊的70例患者BALF标本，根据诊断分为肺癌组（21例，含单纯肺癌14例、肺癌合并感染7例）和非肺癌组（49例，含肺感染性疾病39例、ILD 10例）。采用流式细胞术检测BALF中T淋巴细胞亚群及CD64表达，计算CD4^+^/CD8^+^比值及nCD64指数。采用受试者工作特征（receiver operating characteristic, ROC）曲线评估各指标鉴别肺癌与肺感染性疾病、肺癌与ILD的价值，计算曲线下面积（area under the curve, AUC）及其95%置信区间（confidence interval, CI），并分析CD4^+^/CD8^+^比值及nCD64指数联合对肺癌与肺感染性疾病的鉴别诊断效能。采用Firth's logistic回归进行多因素分析，探讨两指标对肺癌与肺感染的独立鉴别价值。** 结果** 与非肺癌组相比，肺癌组CD4^+^ T细胞比例、CD4^+^/CD8^+^比值及nCD64指数均显著降低（均P<0.05）。进一步分析发现，与肺感染性疾病组相比，肺癌组上述指标亦显著下降（P<0.05），但与ILD组相比无显著差异（P>0.05）。ROC曲线显示，CD4^+^/CD8^+^比值鉴别肺癌和肺感染性疾病的AUC为0.712（95%CI: 0.575-0.849, P=0.008），灵敏度为56.76%，特异度为85.00%；nCD64指数鉴别肺癌与肺感染性疾病的AUC为0.677（95%CI: 0.539-0.814, P=0.026），灵敏度为44.74%，特异度为95.24%。联合应用CD4^+^/CD8^+^比值及nCD64指数时，并联试验的灵敏度提升至78.38%，串联试验的特异度及阳性预测值均达100.00%。校正年龄后，Firth's logistic回归显示CD4^+^/CD8^+^比值和nCD64指数仍对肺癌与肺感染性疾病的鉴别具有独立价值（均P<0.05）。** 结论** BALF中CD4^+^/CD8^+^比值与nCD64指数联合检测可提高肺癌与肺感染性疾病的鉴别诊断效能。

## Abstract (en)

**Background and objective** Distinguishing lung cancer from pulmonary infection and interstitial lung disease (ILD) remains challenging. This study aimed to evaluate the diagnostic value of T lymphocyte subsets and neutrophil CD64 (nCD64) index in bronchoalveolar lavage fluid (BALF), alone and in combination, to address this challenge.** Methods** BALF was collected between October 2021 and October 2022 from 70 patients admitted to West China Hospital, Sichuan University. Based on final diagnosis, the patients were divided into the lung cancer group (21 cases, including 14 with pure lung cancer and 7 with lung cancer and infection) and the non-lung cancer group (49 cases, including 39 with pulmonary infection and 10 with ILD). Flow cytometry was used to detect T lymphocyte subsets and CD64 expression in BALF, and the CD4^+^/CD8^+^ ratio and nCD64 index were calculated. Receiver operating characteristic (ROC) curves were used to evaluate the performance of both indicators in differentiating lung cancer from pulmonary infection and from ILD, and the area under the curve (AUC) and 95% confidence interval (CI) were calculated. The diagnostic efficacy of combining the CD4^+^/CD8^+^ ratio and nCD64 index for differentiating lung cancer from pulmonary infection was further analyzed. Firth’s logistic regression was performed to adjust for age and to assess the independent differential diagnosis value of the two indicators between lung cancer and pulmonary infection. ** Results** Compared with the non-lung cancer group, the lung cancer group showed significantly lower CD4^+^ T cell proportion, CD4^+^/CD8^+^ ratio, and nCD64 index (all P<0.05). Further analysis revealed that the lung cancer group also had significantly lower values of the above indicators compared with the pulmonary infection group (P<0.05), but no significant differences were observed compared with the ILD group (P>0.05). ROC curve analysis showed that the CD4^+^/CD8^+^ ratio had an AUC of 0.712 (95%CI: 0.575-0.849, P=0.008) for differentiating lung cancer from pulmonary infection, with a sensitivity of 56.76% and a specificity of 85.00%; the nCD64 index had an AUC of 0.677 (95%CI: 0.539-0.814, P=0.026), with a sensitivity of 44.74% and a specificity of 95.24%. When the two indicators were combined, the parallel test increased the sensitivity to 78.38%, while the serial test achieved 100.00% specificity and positive predictive value. After adjusting for age, Firth's logistic regression demonstrated that both the CD4^+^/CD8^+^ ratio and nCD64 index retained independent differential diagnosis value for differentiating lung cancer from pulmonary infection (both P<0.05). **Conclusion** The combined detection of the CD4^+^/CD8^+^ ratio and nCD64 index in BALF improves the diagnostic efficacy for differentiating lung cancer from pulmonary infection.

支气管肺泡灌洗（bronchoalveolar lavage, BAL）是一种通过纤维支气管镜获取远端气道及肺泡表面脱落细胞和生化成分的微创技术。随着流式细胞术的广泛应用，分析支气管肺泡灌洗液（bronchoalveolar lavage fluid, BALF）中的细胞亚群及表面分子的平均荧光强度，已成为辅助诊断和评估肺部疾病预后的重要手段^[1]^。

肺癌作为我国恶性肿瘤发病和死亡的首要原因^[2]^，其早期识别与准确鉴别对改善患者预后至关重要。然而，在临床实践中，部分肺癌患者的影像学表现和临床症状与肺感染性疾病或间质性肺疾病（interstitial lung disease, ILD）高度相似^[3,4]^。当肺癌合并感染时，其表现可被感染掩盖，鉴别诊断往往更为困难^[5]^。因此，探索肺癌局部的免疫特征具有重要临床价值。

在适应性免疫中，T淋巴细胞亚群的动态平衡对评估局部免疫状态具有重要意义。CD4^+^辅助T细胞通过分泌细胞因子诱导免疫应答，CD8^+^毒性T细胞可直接杀伤靶细胞^[6]^。临床上常以CD4^+^/CD8^+^比值作为反映机体免疫调节状态的一项重要指标^[7]^。既往研究^[8]^提示，BALF中CD4^+^/CD8^+^在鉴别肺肿瘤和肺良性疾病中有一定的临床价值，但单一指标存在局限，且无法反映固有免疫状态。

除淋巴细胞外，中性粒细胞也是肺部免疫防御的重要组成部分，中性粒细胞CD64（neutrophil CD64, nCD64）的表达变化可反映局部免疫状态。生理状态下，nCD64呈低表达，感染时可在4-6 h内迅速上调达10倍，对早期感染的诊断具有较高的敏感性和特异性^[9,10]^。近年来，nCD64指数在感染性疾病诊断和鉴别中的价值已得到广泛认可^[11]^。在呼吸系统疾病中，该指数在区分肺结核和肺炎^[12]^、辅助诊断恶性肿瘤患者肺孢子虫肺炎^[13]^以及预测慢性阻塞性肺疾病急性加重期预后^[14]^等方面均显示出应用价值。然而，上述研究多以外周血为样本，基于BALF的研究较少，其在肺癌患者BALF中的表达特征和鉴别诊断价值尚不明确。

联合检测CD4^+^/CD8^+^比值与nCD64指数，可从适应性免疫和固有免疫两个维度综合评价肺部免疫状态。目前国内外尚无研究系统探讨两者联合在肺癌鉴别诊断中的价值。因此，本研究旨在分析不同肺部疾病患者BALF中这两项指标的表达特征，评估其单独及联合应用对肺癌与肺感染性疾病、ILD的鉴别诊断效能，以期为临床提供新的免疫学辅助指标。

## 1 资料与方法

### 1.1 临床资料

本研究回顾性收集2021年10月至2022年10月于四川大学华西医院就诊的70例患者。所有患者均经病理学和/或临床诊断确诊，根据最终诊断将患者分为肺癌组（LC组）与非肺癌组（Non-LC组），其中LC组进一步分为单纯肺癌组和肺癌合并感染组；Non-LC组包括肺感染性疾病组和ILD组（包括单纯ILD和ILD合并感染）。收集患者临床资料及BALF标本进行检测。本研究经四川大学华西医院生物医学伦理审查委员会批准并豁免知情同意。

### 1.2 纳入与排除标准

纳入标准：（1）肺恶性肿瘤：经病理学确诊；（2）ILD：依据影像学特征和/或病理学结果确诊；（3）肺感染性疾病：符合中华医学会呼吸病学分会制定的《中国成人社区获得性肺炎诊断和治疗指南（2016年版）》的诊断标准^[15]^；（4）肺恶性肿瘤合并感染及ILD合并感染：在影像学和/或病理学确诊肿瘤或ILD的基础上，同时满足以下任一条件：①BALF培养检出病原体；②BALF的聚合酶链反应或宏基因组二代测序结果阳性。排除标准：（1）临床资料不完整；（2）入院前已接受抗感染治疗；（3）合并其他部位活动性感染；（4）合并人类免疫缺陷病毒感染或其他免疫缺陷疾病。

### 1.3 BALF收集与处理

依据患者胸部高分辨率计算机断层扫描（high-resolution computed tomography, HRCT）表现选择灌洗部位，将支气管镜远端嵌顿于目标肺段支气管开口处，经活检孔分3-4次注入37 ^o^C灭菌生理盐水（总量100-200 mL），随即以50-100 mmHg负压抽吸回收BALF，回收率不低于30%。获取的BALF经两层无菌纱布过滤去除黏液，滤液于1500 g离心10 min，收集细胞沉淀重悬备用。对于临床怀疑肺部感染的患者，BALF样本同时送微生物学及病毒学检测。

### 1.4 流式细胞术检测

#### 1.4.1 主要仪器与试剂

流式细胞仪为美国BD公司FACSCanto Plus，采用CytoDiff组合试剂（包含CD14-FITC、CD64-PE、CD16-PC5、CD45-APC、CD19-AmCyan、CD3-BV421、CD4-PE及CD8-APC共8种单克隆抗体），同时使用校准微球和质控微球进行仪器质控。

#### 1.4.2 检测方法

取100 μL重悬的BALF细胞沉淀，加入10 μL CytoDiff组合试剂，混匀后室温避光孵育15 min。将样本置于进样架，使用FACSCanto Plus系统进行检测，CXP软件采集数据。采用FlowJo软件进行数据分析，基于白细胞表面抗原表达差异及散射光特征，通过多重逻辑设门策略区分细胞亚群，具体步骤如下：（1）基于前向散射光（forward scatter, FSC-A）和侧向散射光（side scatter, SSC-A）圈选全部细胞（图1A）；（2）以CD45和SSC-A设门，圈选髓系细胞和淋巴细胞（图1B）；（3）以CD14和CD16设门，区分中性粒细胞（CD16^+^CD14^dim^）和单核细胞（CD14^+^CD16^dim^）（图1C）；（4）在中性粒细胞门内分析CD64的表达水平（图1D）；（5）以CD3和CD19设门，区分B淋巴细胞（CD19^+^CD3^dim^）和T淋巴细胞（CD3^+^CD19^dim^）（图1E）；（6）以CD4和CD8设门，将T淋巴细胞分为CD3^+^CD4^+ ^T细胞和CD3^+^CD8^+ ^T细胞（图1F，以下简称CD4^+ ^T细胞和CD8^+ ^T细胞）。分别记录各亚群细胞数及平均荧光强度（mean fluorescence intensity, MFI），计算CD3^+^CD4^+^/CD3^+^CD8^+^比值（以下简称CD4^+^/CD8^+^比值）及nCD64指数。nCD64指数计算公式为：（MFI中性粒细胞_CD64_/MFI淋巴细胞_CD64_）/（MFI单核细胞_CD64_/MFI中性粒细胞_CD64_）。

**图1:**
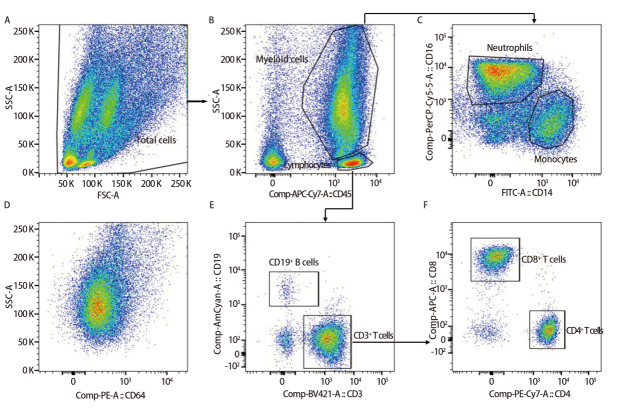
BALF免疫细胞亚群设门策略。A：基于FSC-A和SSC-A圈选全部细胞；B：通过CD45染色设门，区分髓系细胞和淋巴细胞；C：根据CD14和CD16表达，区分中性粒细胞和单核细胞；D：以SSC-A和CD64设门，分析中性粒细胞群中CD64的表达水平；E：通过CD3和CD19染色区分T淋巴细胞与B淋巴细胞；F：通过CD4和CD8染色，识别CD4^+^和CD8^+^ T淋巴细胞亚群。

### 1.5 统计学分析

采用SPSS 26.0软件进行统计分析。计量资料先进行Shapiro-Wilk正态性检验，部分组别数据呈偏态分布且部分组别样本量较小，因此所有计量资料统一以中位数（四分位距）表示；两组间比较采用Mann-Whitney U检验，多组间比较采用Kruskal-Wallis H检验；若差异有统计学意义，进一步采用Bonferroni校正的Mann-Whitney U检验进行两两比较。计数资料以例数（%）表示，组间比较采用卡方检验，当理论频数<5时采用Fisher确切概率法。采用受试者工作特征（receiver operating characteristic, ROC）曲线评估诊断效能，计算曲线下面积（area under the curve, AUC）及其95%置信区间（confidence interval, CI）。多因素分析使用R软件（版本4.4.2）中的logistf包进行Firth’s logistic回归，以校正年龄的混杂效应。因nCD64指数呈偏态分布，分析前取其自然对数转换。P<0.05为差异有统计学意义。

## 2 结果

### 2.1 患者基本特征

2021年10月至2022年10月本研究共纳入70例符合标准的患者，中位年龄为59.00岁（四分位距：51.00-68.00岁），男性42例（60.00%），女性28例（40.00%）。其中，LC组21例，Non-LC组49例。LC组中单纯肺癌14例，肺癌合并感染7例；Non-LC组中肺感染性疾病39例，ILD 10例（含4例单纯ILD和6例ILD合并感染）。

LC组与Non-LC组比较显示，两组患者的年龄存在显著差异（P=0.031），LC组中位年龄（63.00岁）高于Non-LC组（54.00岁）。而在性别（P=0.070）及吸烟史（P=0.223）方面，两组差异均无统计学意义。对各亚组进一步分析发现，四组患者的年龄分布差异显著（P=0.010），其中单纯肺癌组中位年龄最大（63.50岁），ILD组年龄最小（50.50岁）。四组患者的性别构成（P=0.111）及吸烟史（P=0.551）差异无统计学意义。各组患者详细基线特征见表1。

**表1:** 患者基线临床特征

Variable	Total (n=70)	LC group (n=21)				Non-LC group (n=49)				P			
		Pure LC (n=14)	LC with infection (n=7)	LC total (n=21)		Pulmonary infection (n=39)	ILD (n=10)	Non-LC total (n=49)		Among 4 groups	LC vs Non-LC	LC vs Pulmonary infection	LC vs ILD
Gender, n (%)										0.111^c^	0.070^d^	0.129^d^	0.106^c^
Male	42 (60.00)	12 (85.71)	4 (57.14)	16 (76.19)		22 (56.41)	4 (40.00)	26 (53.06)					
Female	28 (40.00)	2 (14.29)	3 (42.86)	5 (23.81)		17 (43.59)	6 (60.00)	23 (46.94)					
Age (yr)										0.010^a^	0.031^b^	0.016^b^	<0.001^b^
Median	59.00	63.50	59.00	63.00		58.00	50.50	54.00					
IQR	51.00-68.00	59.00-69.00	59.00-71.00	59.00-71.00		51.00-68.00	47.50-55.00	50.00-66.50					
Smoking, n (%)										0.551^c^	0.223^d^	0.299^d^	0.280^c^
Former/Current	29 (41.43)	8 (57.14)	3 (42.86)	11 (52.38)		15 (38.46)	3 (30.00)	18 (36.73)					
Never	41 (58.57)	6 (42.86)	4 (57.14)	10 (47.62)		24 (61.54)	7 (70.00)	31 (63.27)					

^a^Kruskal-Wallis test; ^b^Mann-Whitney U test; ^c^Fisher’s exact test; ^d^Chi-square test. LC: lung cancer; Non-LC: non-lung cancer, namely benign pulmonary diseases (pulmonary infection and ILD); ILD: interstitial lung disease; IQR: interquartile range.

### 2.2 BALF中细胞学分类结果

各组患者BALF中淋巴细胞、中性粒细胞及单核细胞百分比的分析结果见表2。LC组与Non-LC组比较显示，LC组中性粒细胞比例显著低于Non-LC组（P=0.004），而两组间单核细胞及淋巴细胞比例均无显著差异（P>0.05）。LC组中性粒细胞比例亦显著低于肺感染性疾病组（P<0.001），但与ILD组的差异无统计学意义（P=0.642）。

**表2:** 各组患者BALF细胞特征比较

Variable	Total (n=70)	LC group (n=21)				Non-LC group (n=49)				P			
		Pure LC (n=14)	LC with infection (n=7)	LC total (n=21)		Pulmonary infection (n=39)	ILD (n=10)	Non-LC total (n=49)		Among 4 groups^a^	LC vs Non-LC^b ^	LC vs Pulmonary infection^b^	LC vs ILD^b^
Lymphocytes (%)										0.173	0.893	0.727	0.118
Median	1.81	1.91	1.39	1.72		1.77	3.07	1.86					
IQR	0.96-3.38	1.50-3.09	0.43-2.07	1.24-2.58		0.66-3.30	1.71-7.44	0.73-3.80					
Neutrophils (%)										<0.001	0.004	<0.001	0.642
Median	66.94	44.05	49.50	45.83		75.31	48.96	71.90					
IQR	45.71-78.44	33.86-57.17	44.08-96.08	35.99-64.05		65.07-82.25	24.42-67.24	57.86-81.05					
Monocytes (%)										0.842	0.445	0.530	0.398
Median	2.09	2.51	4.01	2.63		1.93	1.91	1.93					
IQR	1.30-4.18	1.57-3.33	1.30-5.57	1.44-3.81		1.21-4.62	1.20-5.24	1.21-4.68					

^a^Kruskal-Wallis test; ^b^Mann-Whitney U test.

对各亚组进一步分析提示，中性粒细胞比例在四组间差异显著（P<0.001），肺感染性疾病组中性粒细胞比例最高（75.31%），显著高于单纯肺癌组（44.05%, P<0.05）与ILD组（48.96%, P<0.05）。肺癌合并感染组中性粒细胞比例（49.50%）介于单纯肺癌组（44.05%）与肺感染性疾病组（75.31%）之间，但与两组相比差异均无统计学意义（P>0.05）。淋巴细胞和单核细胞比例在四组间差异均无统计学意义（P=0.173, P=0.842）。

### 2.3 BALF中淋巴细胞亚群分析结果

各组患者BALF中淋巴细胞亚群分析结果见表3。LC组与Non-LC组比较显示，LC组CD4^+^ T细胞比例（P=0.014）及CD4^+^/CD8^+^比值（P=0.011）均显著低于Non-LC组，而CD3^+^ T细胞、CD19^+^ B细胞及CD8^+^ T细胞比例在两组间均无统计学差异（P>0.05）。LC组与肺感染性疾病组比较显示，LC组CD4^+^ T细胞比例（P=0.014）及CD4^+^/CD8^+^比值（P=0.009）亦显著降低。与ILD组相比，LC组CD3^+^ T细胞比例显著降低（66.68% vs 90.71%, P=0.017），而CD4^+^ T细胞比例及CD4^+^/CD8^+^比值在两组间差异均无统计学意义（P>0.05）。

**表3:** BALF中淋巴细胞亚群比较

Variable	Total (n=70)	LC group (n=21)				Non-LC group (n=49)				P			
		Pure LC (n=14)	LC with infection (n=7)	LC total (n=21)		Pulmonary infection (n=39)	ILD (n=10)	Non-LC total (n=49)		Among 4 groups^a^	LC vs Non-LC^b^	LC vs Pulmonary infection^b^	LC vs ILD^b^
CD3^+^ (%)										0.133	0.540	0.786	0.017
Median	70.70	66.68	70.70	66.68		70.02	90.71	71.61					
IQR	50.38-86.63	51.12-84.16	29.38-83.58	46.40-83.58		49.27-85.81	78.15-94.06	50.63-88.08					
CD19^+^ (%)										0.292	0.763	0.365	0.147
Median	3.94	3.79	5.23	3.94		6.39	0.91	3.59					
IQR	1.25-10.75	1.63-4.97	1.85-10.53	1.91-7.06		0.98-16.21	0.62-2.55	0.93-14.52					
CD4^+^ (%)										0.108	0.014	0.014	0.502
Median	38.93	29.84	32.21	32.21		45.83	38.24	43.74					
IQR	27.53-54.00	17.12-43.90	19.15-38.93	25.32-42.06		27.75-56.92	27.45-57.55	27.72-56.92					
CD8^+^ (%)										0.234	0.052	0.050	0.286
Median	48.04	49.58	60.44	60.44		43.68	47.35	44.58					
IQR	32.12-65.18	38.29-66.41	41.61-77.66	41.61-67.64		27.31-60.66	36.02-65.80	29.58-62.27					
CD4^+^/CD8^+^ ratio										0.063	0.011	0.009	0.234
Median	0.77	0.45	0.64	0.51		1.24	0.81	0.93					
IQR	0.43-1.48	0.18-0.91	0.25-0.78	0.25-0.90		0.49-2.15	0.43-1.47	0.46-1.77					

^a^Kruskal-Wallis test; ^b^Mann-Whitney U test.

对各亚组进一步分析显示，肺感染性疾病组CD4^+^ T细胞比例（45.83%）及CD4^+^/CD8^+^比值（1.24）均最高，单纯肺癌组均最低（分别为29.84%和0.45）。肺癌合并感染组CD4^+^ T细胞比例（32.21%）及CD4^+^/CD8^+^比值（0.64）则介于单纯肺癌组和肺感染性疾病组之间，但与两组的差异均无统计学意义（P>0.05）。CD3^+^ T细胞、CD19^+^ B细胞及CD8^+^ T细胞在四组间无显著差异（P>0.05）。

### 2.4 BALF中nCD64指数分析

各组患者BALF中nCD64指数如图2A-2D所示，单纯肺癌组、肺癌合并感染组、肺感染性疾病组及ILD组nCD64指数的中位数（四分位距）分别为：0.22（0.12-0.50）、0.34（0.04-0.47）、0.49（0.16-0.83）和0.43（0.38-1.29）。LC组nCD64指数显著低于Non-LC组（P=0.015）。进一步分析提示，nCD64指数在LC组亦显著低于肺感染性疾病组（P=0.026），但LC组与ILD组相比无明显差异（P=0.059），肺感染性疾病组与ILD组之间亦无显著差异（P=0.713）。肺癌合并感染组的nCD64指数高于单纯肺癌组但仍低于肺感染性疾病组，且与这两组的差异均无统计学意义。

**图2:**
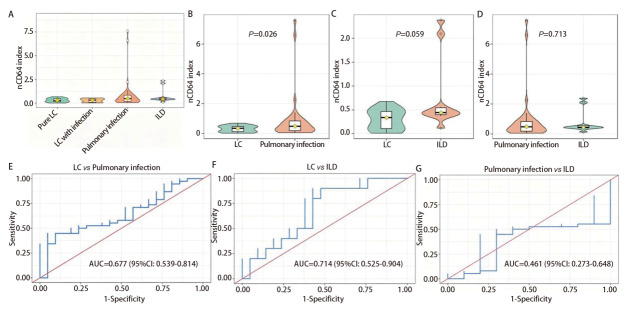
nCD64指数在BALF中的分布及其鉴别肺部疾病的ROC曲线。A：小提琴箱线图展示单纯肺癌组、肺癌合并感染组、肺感染性疾病组及ILD组BALF中nCD64指数的分布；B-D：小提琴箱线图分别展示LC组（n=21）与肺感染性疾病组（n=39）（B）、LC与ILD（n=10）（C）、肺感染性疾病组与ILD组（D）BALF中nCD64指数的水平及组间比较，ILD组包含4例单纯ILD和6例ILD合并感染；E-G：nCD64指数鉴别LC与肺感染性疾病（E）、LC与ILD（F）以及肺感染性疾病与ILD（G）的ROC曲线。

### 2.5 nCD64指数对肺部疾病的鉴别诊断效能

基于上述结果，采用ROC曲线（图2E-2G）评估BALF中nCD64指数在肺部疾病鉴别诊断中的价值，分析结果见表4。nCD64指数区分LC和肺感染性疾病时AUC为0.677（P=0.026），特异度为95.24%，敏感度为44.74%。在区分LC和ILD时AUC为0.714，但无统计学差异（P=0.059）。在肺感染性疾病和ILD的鉴别中nCD64指数效能不佳（AUC为0.461，P=0.713）。

**表4:** nCD64指数鉴别肺部疾病的ROC曲线分析

Group	AUC (95%CI)	Cut-off value	Sensitivity (%)	Specificity (%)	P
LC vs Pulmonary infection	0.677 (0.539-0.814)	0.578	44.74	95.24	0.026
LC vs ILD	0.714 (0.525-0.904)	0.363	90.00	57.14	0.059
Pulmonary infection vs ILD	0.461 (0.273-0.648)	0.577	80.00	44.74	0.713

### 2.6 CD4^+^/CD8^+^比值与nCD64指数联合应用鉴别肺癌与肺感染性疾病的效能

鉴于CD4^+^/CD8^+^比值及nCD64指数在LC组与肺感染性疾病组之间存在显著差异，本研究进一步评估两项指标联合应用在鉴别这两组疾病中的诊断效能。以肺感染性疾病组（39例）为阳性组，LC组（21例）为阴性组，ROC分析结果显示CD4^+^/CD8^+^比值单一检测的AUC为0.712（95%CI: 0.575-0.849, P=0.008），灵敏度为56.76%，特异度为85.00%，截断值为0.958。nCD64指数单一检测的AUC为0.677（95%CI: 0.539-0.814, P=0.026），特异度为95.24%，敏感度为44.74%，截断值为0.578。计算并联试验（两项指标任一阳性即判为阳性）和串联试验（两项指标均阳性方判为阳性）的诊断效能指标，结果见表5。与单独应用相比，并联试验可将灵敏度提升至78.38%，特异度为80.00%，准确度为78.95%，阴性预测值亦提高至66.67%，有助于减少漏诊。串联试验的特异度及阳性预测值均达到100.00%，灵敏度降至21.62%。两种联合策略可根据临床需求（减少漏诊或减少误诊）进行选择。

**表5:** CD4^+^/CD8^+^比值与nCD64指数联合鉴别肺癌与肺部感染的诊断效能

Item	Sensitivity (%)	Specificity (%)	PPV (%)	NPV (%)	Accuracy (%)
CD4^+^/CD8^+^ ratio	56.76	85.00	87.50	51.52	66.67
nCD64 index	44.74	95.24	94.12	48.72	62.50
Parallel test	78.38	80.00	87.88	66.67	78.95
Serial test	21.62	100.00	100.00	40.82	49.12

Parallel test: positive if either CD4^+^/CD8^+^ ratio≥cut-off or nCD64 index≥cut-off; Serial test: positive only if both criteria are met. PPV: positive predictive value; NPV: negative predictive value.

### 2.7 校正年龄后的多因素分析

为排除年龄对免疫指标的潜在影响，采用Firth’s logistic回归进行多因素分析（nCD64指数因偏态分布取自然对数），结果如表6所示。校正年龄后，在LC组与Non-LC组、LC组与肺感染性疾病组的比较中，CD4^+^/CD8^+^比值和ln（nCD64指数）均与Non-LC组和肺感染性疾病组有正向关联（均P<0.05），而年龄无统计学差异（均P>0.05），表明CD4^+^/CD8^+^比值和nCD64指数对肺癌的鉴别价值独立于年龄因素。

**表6:** 校正年龄后Firth’s logistic回归分析

Group	Variable	OR (95%CI)	P
LC (n=21) vs Non-LC (n=49)	Age	0.96 (0.91-1.01)	0.064
	CD4^+^/CD8^+^ ratio	3.68 (1.33-10.20)	0.012
	ln(nCD64 index)	2.48 (1.25-4.91)	0.009
LC (n=21) vs Pulmonary infection (n=39)	Age	0.97 (0.92-1.02)	0.263
	CD4^+^/CD8^+^ ratio	3.66 (1.33-10.10)	0.012
	ln(nCD64 index)	2.31 (1.17-4.57)	0.017

Dependent variable coding: control group=1, lung cancer group=0. The nCD64 index was natural log-transformed due to skewed distribution. OR: odds ratio.

## 3 讨论

多种病原体导致的肺感染性疾病可与肺癌呈现高度相似的临床和影像学特征^[4]^，二者的鉴别诊断仍面临挑战。BALF作为获取肺部局部免疫微环境的窗口，其免疫细胞分析可能为此类鉴别提供新的视角^[16]^。本研究发现BALF中CD4^+^/CD8^+^比值降低与nCD64指数降低分别从适应性免疫和固有免疫两个维度反映了肺癌的局部免疫特征，两者联合应用可显著提升肺癌与肺感染性疾病的鉴别诊断效能。

本研究发现，与肺感染性疾病患者相比，肺癌患者CD4^+^ T细胞比例及CD4^+^/CD8^+^比值均显著降低，提示肺癌局部微环境呈现适应性免疫抑制状态，有利于形成肿瘤快速生长和发生远处播散的环境，这一结果与既往研究^[8,17]^报道一致。有研究^[18]^提示吸烟可导致BALF中CD4^+^/CD8^+^比值倒置，本研究中各组患者吸烟史无显著差异，提示观察到的免疫差异主要源于疾病本身而非吸烟。然而，这种免疫抑制特征在不同类型的肺部良性疾病中表现并不一致。

与ILD组相比，LC组CD4^+ ^T细胞比例及CD4^+^/CD8^+^比值呈下降趋势但无统计学差异，这可能与ILD组样本量较小（n=10）且部分合并感染有关。此外，肺癌合并感染组（n=7）CD4^+ ^T细胞比例及CD4^+^/CD8^+^比值虽高于单纯肺癌组，但仍低于肺感染性疾病组，与两组无显著差异，这一结果同样可能受到该亚组样本量较小的限制，结果需要谨慎解读。从趋势上看感染可一定程度上改变肺癌局部免疫微环境，但尚不足以完全逆转肿瘤相关的免疫抑制特征。因此，本研究发现CD4^+^/CD8^+^比值下降仍是鉴别肺癌与肺感染性疾病的重要指标。

在固有免疫层面，nCD64作为中性粒细胞活化标志物，在感染鉴别中具有高特异度，已被证实可有效区分感染性与非感染性疾病^[19,20]^。近年来，国内专家共识已明确推荐采用标准化nCD64指数（以单核细胞和淋巴细胞分别作为阳性和阴性对照）进行检测，使研究结果更加客观可靠^[11]^。本研究中肺感染性疾病组nCD64指数显著高于LC组，与上述生物学特性一致，提示中性粒细胞在肺部感染中发挥重要的免疫功能。当肺癌患者合并感染时，nCD64表达水平介于单纯肺癌组与肺感染性疾病组之间，提示伴发感染可部分激活肺癌局部的固有免疫应答，但其激活程度尚不足以完全改变肿瘤背景下的免疫特征。

在鉴别肺癌与肺部感染时，nCD64指数表现出高特异度，提示若BALF中nCD64指数升高超过截断值，感染的可能性极大，有助于避免将感染误判为肺癌而延误抗感染治疗。然而，nCD64指数的灵敏度较低，单独应用时漏诊风险较高，因此不宜作为排除感染的单一指标。在肺癌与ILD、ILD与肺感染性疾病的鉴别中，nCD64指数未显示出显著价值，这可能与ILD组样本量较小（10例）且部分合并感染有关，有待扩大样本量进一步验证。

CD4^+^/CD8^+^比值以及nCD64指数从不同维度评估了肺部局部免疫状态，二者在肺癌和肺感染性疾病的鉴别诊断中都呈现出一定的价值和局限。基于此，本研究进一步评估了两项指标联合应用的诊断效能。与单独应用相比，并联试验将灵敏度提升至78.38%，而串联试验的特异度及阳性预测值均可达100.00%。两项指标的互补性可能源于其分别反映适应性免疫和固有免疫的不同维度。基于本研究结果，并联试验在提高灵敏度方面显示出潜力，提示其在筛查场景中可能有助于减少漏诊；串联试验在提高特异度方面显示出优势，提示其在确诊场景中可能有助于避免误诊。为排除年龄对免疫指标的潜在影响，本研究采用Firth’s logistic回归进行多因素分析。结果显示，校正年龄后，CD4^+^/CD8^+^比值和nCD64指数仍与肺感染性疾病显著关联（均P<0.05），而年龄无统计学差异。这表明两项指标对肺癌与肺感染性疾病的鉴别价值独立于年龄因素，进一步支持了本研究结论的稳健性。

本研究具有一定的局限性：（1）样本量较小，尤其是肺癌合并感染组和ILD组，可能导致统计效能不足，部分结果需谨慎解读；（2）ILD组包含合并感染病例，且由于样本量限制，未能做亚组分析，可能影响对ILD本身免疫特征的分析；（3）免疫指标覆盖不全[未检测调节性T细胞（regulatory T cells, Treg）、自然杀伤（natural killer, NK）细胞及相关细胞因子]，且依赖流式细胞术检测，对免疫微环境的评估尚不全面。尽管存在上述局限，本研究的核心发现，即CD4^+^/CD8^+^比值和nCD64指数在肺癌与肺部感染鉴别中的价值，在主要比较组（肺癌 vs 感染）中仍具有统计学意义，提示结论具有一定稳健性。

综上所述，BALF中CD4^+^/CD8^+^比值与nCD64指数联合检测可有效提高肺癌与肺感染性疾病的鉴别诊断效能，并联试验和串联试验可根据临床需求进行选择，具有潜在的临床应用价值，但后续需开展多中心前瞻性研究，扩大样本量并纳入更多免疫指标，进一步验证本研究的结论。
